# Supplementing Colostrum from Multiparous Sows: Effects on Performance and Health in Piglets from Gilts in Farm Conditions

**DOI:** 10.3390/ani11092563

**Published:** 2021-08-31

**Authors:** Joaquin Miguel, Olga Mitjana, María Teresa Tejedor, Antonio Martínez, María Victoria Falceto

**Affiliations:** 1 Department of Animal Pathology, University of Zaragoza, C/Miguel Servet 177, 50013 Zaragoza, Spain; jmiguelescuder@gmail.com (J.M.); vfalceto@unizar.es (M.V.F.); 2 Agroalimentary Institute of Aragon-IA2, University of Zaragoza-CITA, C/Miguel Servet 177, 50013 Zaragoza, Spain; 3 Department of Anatomy, Embryology and Animal Genetics, University of Zaragoza, C/Miguel Servet 177, 50013 Zaragoza, Spain; ttejedor@unizar.es; 4 CIBER CV, Faculty of Veterinary Medicine, University of Zaragoza, C/Miguel Servet 177, 50013 Zaragoza, Spain; 5 Vall Companys, Polígono Industrial Valdeferrín, 50600 Ejea de los Caballeros, Spain; agilaberte@vallcompanys.es

**Keywords:** colostrum, multiparous sows, gilts, piglets’ performance

## Abstract

**Simple Summary:**

Colostrum intake is essential for piglets. Gilt litters may not receive the same quantity and quality of colostrum as the litters from sows do. An extra dose of 30 mL divided into two doses (20 min apart, using a gastric tube) of colostrum from multiparous sows was administered to piglets born from gilts to ascertain its effects on piglets’ performance and health in farm conditions, with a special interest in the smallest piglets (under quartile 1, Q1). Quartiles for birth weight were Q1 = 1.100 kg, Q2 = 1.300 kg, and Q3 = 1.500 kg (*n =* 401). The control group (CON) consisted of 200 piglets from 18 gilts (50 smallest piglets), and 201 piglets from 16 gilts (52 smallest piglets) formed the supplemented group (SUP). Colostrum supplementation increased the homogeneity of weight and average daily gain (ADG) and decreased the use of antibiotics and mortality by diarrhoea. Immune response improved among SUP piglets for the diseases evaluated. In the smallest piglets, colostrum supplementation had significant effects on mean weight and ADG in the first days of life. The smallest piglets had a reduced use of antibiotics when supplemented. The time and labour invested in colostrum supplementation could be compensated by the improvement of piglets’ productive parameters and health.

**Abstract:**

Gilts produce less colostrum with lower immunoglobulin G concentration than multiparous sows do. An extra dose of colostrum (30 mL) from multiparous sows was administered to piglets from gilts to ascertain its effects on performance and health in farm conditions, especially in the smallest piglets (birth weight < 1.100 kg; Q1). The control group (CON) consisted of 200 piglets from 18 gilts (50 smallest piglets) and 201 piglets from 16 gilts (52 smallest piglets) formed the supplemented group (SUP). Colostrum supplementation increased the homogeneity of weight (days 21 and 60) and average daily gain (ADG; days 0–10, 0–21, and 0–60) and a decreased use of antibiotics and mortality by diarrhoea (*p* < 0.05). SUP piglets showed better immune response (presence of antibodies, *p* = 0.033) against *Mycoplasma hyopneumoniae* (day 21), porcine reproductive and respiratory syndrome (PRRS; day 60), and influenza (day 60). In the smallest piglets, colostrum supplementation had important effects on mean weight in the first day of life (*p* = 0.009) and ADG until day 21 (*p* < 0.05). The smallest piglets had decreased the use of antibiotic treatment use when supplemented (*p* < 0.05). Colostrum supplementation can improve piglets´ performance and health, although doing so requires increased time and labour in maternity.

## 1. Introduction

Colostrum intake is essential for piglet growth and development, in not only the lactation phase [1,2] but also later ages. Piglets that ingest less than 290 g of colostrum have a 15% lighter bodyweight than those that ingest >290 g during nursery [3]. Colostrum intake may have long-term effects on the growth of piglets from 3 weeks of age until after fattening [4]. Colostrum provides piglets with warmth, energy, and the immunity and growth factors involved in intestinal development. It is most accessible during the first 12 h from birth; therefore, no piglet should be moved before this time [5,6]. The colostrum intake among piglets is highly variable, ranging from 0 g to more than 700 g [3]. The highest level of consumption indicates that the piglets’ ingestion capacity is extremely high when the colostrum supply is not restricted [5]. At least 200 g of colostrum per piglet is necessary to keep them alive during the neonatal phase [3]. Although piglets would have difficulties in feeding on large amounts of colostrum, improving the amount of colostrum ingested will increase the essential nutrients provided [7]. There are several ways to increase colostrum intake: increasing the ability of piglets to access a teat, reducing intra-litter weight variation, and increasing the amount of colostrum produced by sows [3].

Sows show great variation in colostrum production and composition [5,8,9]. Colostrum production averages 3.3–3.7 kg postpartum but ranges from 1.5 to 6 kg [1,9,10]. Multiple variation factors affect colostrum production, mainly gestation length, litter weight at birth, and the interval between birth and the first lactation action by the piglets (first suckle) [2]. Sow parity can also play a role. Gilts have less mammary tissue and DNA than multiparous sows do [11], leading to a 21% reduction in colostrum and milk production, as compared to fourth-parity sows [12]. A positive correlation exists between DNA content (indicative of the number of cells) of the mammary glands and the final growth rate in piglets [11].

Colostrum is of great immunological importance for newborn piglets [12] and parity seems to influence antibodies concentration. Gilt colostrum has significantly lower IgG concentrations [13], which decreases more rapidly over the first 24 h [9] when compared with that from sows. Thus, gilt litters may not receive the same quantity and quality of colostrum as litters from sows do; the parity of the sow can be an important factor influencing the growth and survival of piglets [14]. With an adequate colostrum intake, IgG is transferred through the epithelial barrier and into circulation, which provides passive immunity to the new-born piglets [13,15,16]. Piglets acquire IgG from the colostrum by absorption through the intestine in the first 24 to 36 h after birth, at which time transport ceases [17,18,19]. Therefore, good maternal immunity can only be achieved by adequate colostrum intake by piglets [3,8,20].

In commercial practice, supplementary feeding typically includes administering colostrum, milk replacers, or glucose to weak piglets [5,21]. The success of these techniques will depend on the worker’s skill [22]. Differences in colostrum intake could explain the variation in pre-weaning mortality among herds [23]. Therefore, although increasing colostrum intake is costly, evidence exists that it can be economically compensated for by improving the piglets’ productive parameters [2]. In this work, we administered an extra dose of colostrum (30 mL) from third- to sixth-parity sows to piglets born from gilts, with two objectives: (1) to determine its impact on growth, mortality, and use of antibiotics among all these piglets in the conditions of a commercial farm and (2) to study, in these conditions, its effect on growth, mortality, and use of antibiotics among piglets below the first quartile (Q1) in birth weight (the smallest piglets). We hypothesized that given multiparous sows seem to produce more abundant and better-quality colostrum than gilts do; therefore, the colostrum supplementation of piglets from gilts with colostrum would improve their performance and health.

## 2. Materials and Methods

This study complied with the European laws on the protection of pigs and animals used for scientific purposes (Council Directive 2008/120/EC outlining minimum standards for the protection of pigs and Directive 2010/63/EU of the European Parliament and of the Council of 22 September 2010 on the protection of animals used for scientific purposes) and the ARRIVE guidelines [24]. All procedures in these experiments were performed in accordance with the precepts of animal welfare and approved by the Committee of Ethics in Animal Experimentation of Universidad de Zaragoza (protocol no. PI08/21).

### 2.1. Animal Management and Experimental Design

#### 2.1.1. Gilts and Piglets

This trial was carried out on a 2500-sow farm in Zaragoza, Spain, in summer (the last two weeks of July). In the present study, 35 crossbred gilts (Large-White x Landrace) were included and bred with Pietrain males.

These gilts were moved to the farrowing area at 111 days of gestation and were housed in individual farrowing crates (2.2 × 2.6 m). The gilts had free access to water and were fed twice daily until day 10 after farrowing with up to 6 kg of lactation feed (Net Energy, NE: 2300 kcal/kg). From day 10 onwards, they were fed three times a day, with the quantity of feed increasing daily according to the farm protocols (up to 10 kg). Throughout the experiment, the gilts were checked daily for health, eating, and other problems.

The gilts (*n* = 35) were divided randomly into two groups (*n* = 17 and *n* = 18, respectively). The piglets of 18 gilts were not supplemented with colostrum and only received colostrum from their mothers (control group: CON group) and that the piglets of 17 gilts were fed colostrum by their mothers at least once, which was supplemented with 30 mL of colostrum previously collected from multiparous sows from third to sixth parity (colostrum supplement group: SUP group). All piglets were supervised in the first 24 h after birth, to ensure their maternal colostrum intake. A total of 401 piglets were included in the trial (CON group: *n* = 200; SUP group: *n* =201). The piglets in each group were weighed and individually identified with ear tags specific for the group when:

- the gilts had given birth to 14 piglets or
- farrowing was finished and the number of piglets was less than 14.

**Figure 1:**
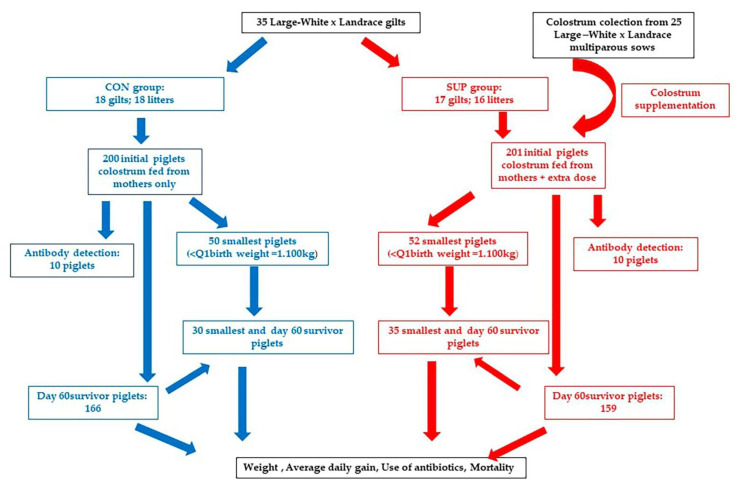
Study design. CON: control group; SUP: colostrum supplemented group. See Material and Methods for details. Weight and average daily gain were only analysed in day 60 survivor piglets.

To reduce the effect of the variation in the number of piglets born, all the gilts in the study were cross-fostered to 14 piglets within 24 h after birth. Gilts with fewer than 14 piglets were equalized with piglets randomly selected that were not identified with the ear tags of the trial, and those with more than 14 piglets also were equalized to 14 piglets. If a gilt in the trial had more than 14 piglets, the excess was moved to other sows. No criteria related to sex or weight were followed in that case. All of the cross-fostering management occurred within 24 h after birth, once all of the piglets had gotten the colostrum from their mothers. The birthdate, number of piglets born alive, and stillborn, and mummified piglets were recorded after the farrowing was completed, as was any extraordinary management (additional veterinary care due to delivery or perinatal problems). Birth weight quartiles were calculated (Q1, Q2, and Q3). Performance and health of piglets in the lowest quartile (<Q1) were further studied (see Section 2.1.3) until 60 days of age. Figure 1 shows the study design.

#### 2.1.2. Piglet Management

The management, actions, and treatments during lactation and the post-weaning period carried out for piglets from both the CON and SUP groups were under commercial conditions as usual. All piglets in the study were supervised to ensure that received colostrum from their mothers in the first hours after farrowing.

At 3 days of age, the piglets were injected intramuscular iron (Ferrovall^®^, Mevet SA, Lleida, Spain; 2 mL) and oral coccidiostatic (Cocciklass^®^, Mevet SA, Lleida, Spain; 2 mL) and were tagged on the other ear with the usual ear tag. From the tenth day after farrowing until the weaning day, creep feed was available for the piglets (100 g per day and litter). On the weaning day (day 21), the piglets were vaccinated against *Mycoplasma hyopneumoniae* and Porcine Circovirus type II (Flexcombo Ingelvac Mycoflex and Circoflex^®^, Boehringer Ingelheim Vetmedica, Duluth, GA, USA).

The piglets were transferred to the post-weaning area at 23 days of age (±2 days). The pens’ area was approximately 9 m^2^ and housed about 40 piglets each, with a covered part and exit to an outdoor area. On the inside, the floor was made of concrete and was heated. On the outside, it was gridded. Two hoppers with six mouths each were available on the inside and two water points with nipple drinkers on the outside. Piglets from both groups were randomly allocated into eight pens and fed according to the farm’s nutrition-feeding program (5–6 kg of pre-starter and 15–20 kg starter feed per piglet during all of the nursery period). At 9 weeks of age (±2 days), every piglet was transferred to the fattening area.

#### 2.1.3. Measures on Piglets

Piglets from both groups were weighed using a 100 kg MS weighing scale (MS Schippers, Barcelona, Spain) at different moments: on the day of birth (day 0), and 1, 10, 21, and 60 days of age. The accuracy of the scale was ±50 g. Prior to each weighing control, it was calibrated according to the manufacturer´s procedure. The average daily gain (ADG) was calculated between each weighing period. The use of antibiotics was measured as the percent of piglets receiving injectable antibiotic treatments at least once. General mortality (global and by age) and mortality by cause were recorded from the day of birth to 60 days of age.

Ten piglets from both the CON and SUP groups were randomly selected for blood sample collection at 21 days of age (prior to vaccination) and 60 days of age, using sterile Biochemistry serum tubes Venoject^®^ (VT-100STK, Terumo Corporation, Tokio, Japan). Blood serum was obtained after coagulum removal, and serum was stored at −20 °C until being sent to the GSP laboratory (Grup de Sanejament Porci, Lérida, Spain) for analysis. The presence of antibodies against porcine reproductive and respiratory syndrome (PRRS), influenza, and *Mycoplasma hyopneumoniae* were detected by means of IDEXX PRRS X3^®^ (IDEXX Laboratories, Inc. Westbrook, ME, USA), CIVTEST SUIS INFLUENZA^®^ (HIPRA, Amer, Gerona, Spain) and CIVTEST SUIS MYCOPLASMA^®^ (HIPRA, Amer, Gerona, Spain), respectively. The reference values for these techniques were as follows:

- IDEXX PRRS X3: Values S/P ratio < 0.4 are considered negative; S/P ≥ 0.4 are considered positive. (S/P, Sample to Positive ratio)
- CIVTEST SUIS INFLUENZA: Values IRPC < 20 are considered negative; IRPC ≥ 20 are considered positive. (IRPC, Relative Index ×1000)
- CIVTEST SUIS MYCOPLASMA: Values above 30% OD are considered positive. (OD, Optical density)

#### 2.1.4. Collection of Colostrum from Multiparous Sows

During the week prior to farrowing of the study gilts, colostrum was collected from 25 multiparous sows (third to sixth parity). Colostrum was collected randomly from multiparous sows that were third to sixth parity and had just given birth. No colostrum was collected from sows with a high number of piglets, in any case, to ensure that all their piglets had their colostrum needs met. The colostrum collection was carried on teats not occupied by any piglets and consequently, the feeding behaviour of their offspring was not disturbed. The maximum volume collected was 250 mL (average volume of colostrum to be ingested by a piglet). To enhance the colostrum ejection, 2 mL of oxytocin (Oxipartvall^®^, Mevet SA, Lleida, Spain) was administered intramuscularly [10] and colostrum was collected from the mammary glands at both sides (average duration of collection: 5 min). A total of 250 mL of colostrum was collected per sow and every individual’s colostrum was pooled to avoid individual variation and stored in 250 mL bottles at −20 °C [2]. A total of 6.50 L was collected from 25 sows. No test about antibody concentration was carried out on the pooled colostrum.

#### 2.1.5. Supplementation with Colostrum of Piglets in the SUP Group

One hour before the supplementation, the bottles containing colostrum were placed in a water bath at 39 °C; at this temperature, the nutritional, immune, and energy qualities of colostrum do not change [19]. No changes in appearance or consistency were noticed and every bottle was gently homogenized before the administration to the piglets.

Once the farrowing of gilts in the SUP group finished and their colostrum was fed to their piglets, the weighed and tagged piglets received colostrum supplementation from multiparous sows. No supplementation was administered to piglets in the CON group, which only received colostrum from their mothers. Every piglet received two 15 mL doses, 20 min apart, due to the small size of the piglets’ stomachs at birth [19]. The colostrum was administered intragastrically. A post-cervical insemination probe (Magaplus S^®^, Magapor S.L., Ejea, Spain) was cut to a 20 cm-length, leaving the tip of the probe at the end to avoid harming the piglets’ digestive tract. The technique gavaging described by Campbell et al. [25] was followed. The piglets were held by the head with their lips opened with the index and thumb fingers. Then, the probe was introduced immediately through the oesophagus to the stomach; 15 mL of colostrum was administered with a syringe in a few seconds, as the colostrum was placed directly in the stomach.

### 2.2. Statistical Methodology

The statistical analysis was carried out using the IBM SPSS Statistics 22.0 software package (SPSS, Chicago, IL, USA). In all of the comparisons, a difference was considered statistically significant when *p* < 0.05 (level of significance α = 0.05). The quartiles (Q1, Q2, and Q3) for weight at birth were calculated from the total number of piglets considered (*n* = 401).

The arithmetic mean, standard deviation (SD), and coefficient of variation (CV = SD/mean) were calculated for each quantitative variable (weight and ADG). The variances of both groups were compared using Levene’s test. The ANCOVA (analysis of covariance) was used to determine the effects of group and litter on weight and ADG after adjusting/controlling for one or more continuous variables (covariates). The group (CON or SUP) was considered a fixed factor, and the litter was considered a hierarchical random factor with respect to the group. Since weight on a particular day of life would be influenced by the weight on previous days, previous measures were considered as covariates when studying piglets’ weight. Additionally, initial weight at the beginning of time intervals for ADG would influence ADG values; therefore, these initial weights were considered as covariates for ADG analysis.

For the qualitative variables (percent of injectable antibiotic treatment, global mortality, and mortality by pathological cause), the proportions were compared between groups using Pearson’s chi-squared (with continuity correction for 2 × 2 tables). Fisher’s exact test was used when any of the cells in the 2 × 2 tables presented an expected number of less than 5 (antibody presence or mortality by diarrhoea). Bonferroni correction was used for multiple comparisons between cells within rows.

More details on the statistical calculations and the tests used are available in Petrie and Watson [26].

## 3. Results

### 3.1. Reproductive Performance of Gilts

In total, 34 litters were counted (18 in the CON group and 16 in the SUP group) since one gilt in the SUP group arrived empty at the maternity area. The average duration of gestation was 115.2 ± 0.18 days. No significant differences were found for the reproductive parameters in both groups (*p* > 0.05) (Table 1).

### 3.2. Evolution of Piglet Weight and ADG

From the 201 and 200 piglets initially assigned to the CON and SUP groups, respectively, only 159 and 166, respectively, survived until the end of the experiment (day 60); therefore, weights and ADG were only analysed in these surviving individuals.

As shown in Table 2, significant differences were found between groups for weight variances: piglets in the SUP group showed higher weight homogeneity than those in the CON group did at the end of the lactation period (day 21; *p* = 0.005) and the last phase of the nursery period (day 60; *p* = 0.012). Litter had a significant effect on weight mean at every time point (*p* < 0.001); also, significant effects of previous measures were detected, especially for the most recent previous weighings (*p* < 0.001). No significant effect of group on weight means was found (*p* > 0.05).

Table 3 shows the ADG data. The highest CV values corresponded to days 0–1. Significant differences between groups were found for ADG variance in the days 0–10 (*p* = 0.038), 0–21 (*p* = 0.003), and 0–60 (*p* = 0.026) periods; therefore, piglets supplemented with multiparous colostrum had more homogeneous ADG values throughout the lactation and weaning phases. Litter had a significant effect on ADG mean at every time interval (*p* < 0.001); also, significant effects of initial weight were detected at most of the considered periods (*p* < 0.001), with the effect of birth weight being especially relevant. No significant effect of the group on ADG means was found (*p* > 0.05).

The quartiles for birth weight were Q1 = 1.100 kg, Q2 = 1.300 kg, and Q3 = 1.500 kg (*n =* 401). Of the piglets, 52 and 50 piglets in the CON and SUP groups were under Q1, respectively. Only 35 piglets in the SUP group and 30 piglets in the CON group survived until the end of the post-weaning phase (day 60th). Table 4 and Table 5 show the weights and ADG of these survivor individuals.

No significant differences were found between groups in weight variance (Table 4). Mean weight was significantly greater for the SUP group on day 1 (*p* = 0.009). Litter had a significant effect on weight mean only for day 60 (*p* = 0.006); also, significant effects of previous measures were detected, especially for the most recent previous weighings (*p* < 0.01).

As shown in Table 5, no significant differences between groups were found for ADG variance (*p* > 0.05). Significant differences were found between groups for mean ADG until day 21 (*p* < 0.05); the SUP group showed greater values. Litter had a significant effect on mean ADG only for the days 0–60 and 21–60 periods (*p* < 0.01). Additionally, a significant effect of initial weight was detected for the days 0–60, 1–10, and 10–21 periods (*p* < 0.05); therefore, the weight of the piglets at the beginning of the periods 0 to 60, 1 to 10 and 10 to 21 days had a significant influence on the piglets’ grown throughout those periods.

### 3.3. Use of Injectable Antibiotic Treatment

Table 6 shows the percentages of injectable antibiotic treatment in both groups, The SUP group had a lower percentage of individuals receiving injectable antibiotic treatment (*p* < 0.05). No significant differences were found between groups when the treatment period was considered (lactation, nursery, or both; *p* = 0.373).

Table 7 shows the results for percentages of injectable antibiotic treatment among piglets under Q1 birth weight. This variable showed higher values than in Table 6, but significant differences between groups were found for initial piglets, with better indices for the SUP group (*p* < 0.05).

Table 8 shows the distribution of diseases observed in the initial piglets. Significant differences were detected between groups (*p* = 0.017). Diarrhoea was the more frequent disease in the studied farm (57.40% of total disease in piglets until day 60). The percentage of diarrhoea-affected piglets was higher in the CON group, while the percentage of thin piglets was higher in the SUP group. No difference was found for polyarthritis.

Diarrhoea affected 33.3% (9/27) and 68.6% (24/35) of piglets under Q1 birth weight in the SUP and CON groups, respectively (*p* = 0.012).

### 3.4. Antibody Detection

Table 9 shows the presence of antibodies against PRRS, influenza, and *M. hyopneumoniae* in 10 individual blood samples from the two groups. Higher percentages of positive samples were detected from the SUP group for PRRS and influenza (day 60; *p* = 0.033) and for *M. hyopneumoniae* (day 2; *p* = 0.033). Samples from the CON group were always negative, except for one piglet, which showed antibodies against influenza at day 21. No antibodies against M. *hyopneumoniae* were detected at day 60.

### 3.5. Mortality and Cause of Death

Table 10 shows the mortality data. General mortality was considered globally, and mortality by age (lactation or nursery period) was estimated. Mortality by cause of death was evaluated, specifically mortality by pathological cause (excluding accidental deaths), both globally and by age (lactation and nursery periods). No differences were found between both groups in any case *(p* > 0.05). Diarrhoea was the more frequent mortality cause in the studied farm (26.30% of total mortality in piglets until day 60); mortality by diarrhoea was more frequent in the CON group than in the SUP group (*p* = 0.037).

Table 11 shows the mortality data for the piglets under Q1 birth weight. No significant differences were found between groups (*p* > 0.05).

## 4. Discussion

There is a lack of information about nutritional strategies for new-born piglets, especially with regard to oral supplementation with colostrum and the results of this practice [27]; therefore, comparing our results with those of previous studies is difficult, particularly for piglets born to gilts. Another way of ensuring adequate colostrum intake by piglets is through supplementation with foreign sow colostrum [28,29]. Commercial bovine colostrum has also been tested in the first-week post-weaning of piglets with a positive effect on the early colonization of the gastrointestinal microbial community. Thus, the number of potential pathogenic enterotoxigenic *Escherichia coli* detected was lower than compared to piglets fed a milk replacer (from bovine milk) [30].

It should be noted that the administration of an additional 30 mL of liquid in the SUP group could cause substantial gastric distension with respect to the absence of distension in the CON group. This fact could influence the function of the stomach and duodenum (motility, secretion, local blood flow, etc.). Around 20–25 min was required to administer 30 mL colostrum divided into two 15-mL doses to 14 piglets (without taking into account the time between the first and second dose). In view of obtained results, the time and labour invested in colostrum supplementation could be compensated by the improvement of piglets´ productive parameters and health.

On the other hand, previous studies have reported that macronutrient levels and total immunoglobulin concentrations in colostrum and sow milk vary by breed and are influenced by many factors, such as perinatal feeding strategy, body condition, parity number, vaccination planning, endocrine control, the health status of the mammary gland and environmental conditions [31,32,33].

### 4.1. Evolution of Piglet Weight and ADG

Piglet performance depends on the lactating sow’s characteristics, which limit piglets’ genetic potential for weight gain [34]. Piglets with a similar weight at cross-fostering (on average 1.4 kg) had a lower daily weight gain (200 g vs. 250 g) when they were adopted by gilts, as compared to sows at fifth parity [35]. Differences in litter performance by sow parity could be minimized using colostrum-enhancing techniques such as additional administration of colostrum from multiparous sows [36].

Supplementation with colostrum from multiparous sows did not show a demonstrable effect on weight and ADG means. Weight and ADG depend on multiple variants, as shown by ANCOVA, with litter and birth weight (especially in early life) having the most important effects. These results agree with those of previous studies [37,38,39,40].

However, this supplementation led to increased homogeneity in both weight and ADG, thus reducing the variability of final weight and daily growth. Piglets in the SUP group showed more homogenous weight and growth at the end of the post-weaning phase (day 60). This suggests that management efforts to improve the amount of colostrum ingested by neonatal piglets would benefit production efficiency [29]. These results agree with previous works suggesting that colostrum intake may have long-term effects on the growth of 3-week-old piglets until after weaning [3]. However, in IUGR (Intrauterine growth retardation) piglets were administered both a bolus of colostrum and an external source, the positive effect on blood glucose levels, rectal temperature, and growth was maintained only during the first 4 h [41].

On the other hand, in the first 24 h after birth, several piglets suffered weight loss while others experienced weight gain. These facts would explain the highest CV values corresponding to ADG at days 0–1. Previously, Foisner et al. [10] reported that ADG in this period differed significantly among litters.

The advised care of piglets with low weight at birth includes assistance to a teat, divided lactation, and supplementary feeding, which may be necessary before or after adoption [42,43]. Piglets under Q1 birth weight that were supplemented showed greater mean weight at day 1 and mean ADG until day 21. Thus, colostrum supplementation seems to have a greater impact on these variables for the smallest piglets than for piglets in general. These smallest piglets show reduced energy reserves and capacity for body temperature maintenance; therefore, they need more time to reach the udder and have difficulty reaching the most productive nipples [44]. This supplementation might provide them with not only early extra energy input but also valuable immunological protection, giving the obtained results; such protection will result in comparative advantage, thus helping to reduce mortality in low-birth-weight piglets born from primiparous sows [45]. However, Viollot et al. [41] did not find clear positive effects with the supplementation of colostrum and oral protein-energy supplement administrated both or separately in low-birth-weight piglets (between 804–1309 g). Pre-weaning mortality was not reduced and only a marginal weight gain was detected in the supplemented group.

### 4.2. Use of Injectable Antibiotic Treatment

Supplementation with colostrum from multiparous sows decreased antibiotic use, especially during the nursery phase. Colostrum provides new-born piglets with passive immunity [20]. Immune status at weaning is influenced directly by the extent of passive immunity gained through colostrum intake [3]. Carney-Hinkle et al. [36] suggested that progeny from gilts could have worse health due to acquiring immune protection through colostrum/milk. A 15-mL supplement of colostrum ensures proper IgG levels in piglets on the fourth day of life [39]. Therefore, our good results regarding antibiotic use could have resulted from the higher immune level in piglets receiving higher-quality colostrum from multiparous sows. Ensuring colostrum intake is a recommended strategy to prevent enteritis and thereby decrease antibiotic treatment [43,46]. Our results proved the success of this strategy. Antibiotic use percent were higher for the smallest piglets than for piglets in general, pointing to the former’s increased vulnerability. However, colostrum supplementation improved these variables. Muns et al. [45] found similar results.

### 4.3. Antibody Detection

Few piglets were sampled, but the significant differences found can be considered a hopeful first approximation. Piglets are born immunologically naïve because a sow is unable to transfer antibodies in utero to piglets via the placenta [5]; thus, antibody transfer from colostrum is crucial for adequate immune function [47]. Maternal antibodies play an important role in the neonatal protection of piglets from infectious agents in the immediate postnatal days before they can develop robust immunities of their own. The vaccination status of the sow and immune difference between sows influence the level of colostrum IgG [48,49].

The parity of sows affects the passive transfer of immunity to piglets; multiparous sows show increased IgG concentrations in serum, milk/colostrum, and piglets´ serum than gilts and their progeny do [9,13,36,50]. Colostrum begins to form in the sow about one month before farrowing, with an intense transfer of immunoglobulin from the serum to the udder. This process will cease after farrowing, decreasing the concentration of IgG in the colostrum [51]. The IgG concentration in colostrum varies significantly between sows and decreases by approximately 80% between 0 and 24 h post-parturition [10]. IgG concentrations in piglet plasma at 24 h of age are strongly correlated with colostrum intake [1,51].

Parity also influences the metabolomics profile of colostrum; lactose creatine, myo-inositol, and O-phosphocholine partially explain the colostrum’s IgG Brix percentage [14]. Through IgG Brix percentage, these metabolites related to a sow’s metabolic condition can influence litter weight at birth and piglets’ mortality; the parity of the sow can be an important factor influencing the growth and survival of piglets [14]. Absorption of IgG through the intestines of new-born piglets could be mediated by a specific receptor, the neonatal fragment crystallisable receptor (FcRn), as in ruminants. The mammary glands and intestines of adult pigs express FcRn [15,16]. Cabrera et al. [19] data indicated a down-regulation of receptor FcRn at 24 h postpartum. In summary, the highest absorption of IgG occurred when the piglets took colostrum immediately after birth. In the current context of reducing antibiotics, improving the immune system would lead to less use.

A positive correlation was found for plasma IgG concentration at the days of age 1 or 2 and at weaning [20]; therefore, it appears that the degree of passive immunity shortly after birth affects the piglets’ immune status at weaning [1]. Direct stimulation of active immunity by passive immunity of the piglet has not yet been demonstrated, and it is difficult to determine the proportion of the present IgG in the plasma at weaning that comes from colostrum versus the proportion produced by the piglet [3]. However, the strong association between colostrum IgG and piglet IgG shows that increased IgG levels in the colostrum will improve the IgG levels in piglets and potentially improve piglets’ survival [52].

### 4.4. Mortality and Cause of Death

Muns et al. [39] did not find an effect of oral colostrum supplementation on litter mortality. However, Viehmann et al. [53] detected improved survival during the first 10 days of life in piglets supplemented with bovine colostrum (1 mL on days of life 1–3).

The temperature inside the maternity wards was elevated during the experiment, which would explain the high general mortality in the maternity wards for the studied piglets (66/401; 16.5%); a similar value was found for the whole farm in summer, while the annual average value for general mortality was only 11%. Both groups had high numbers of crashed piglets (22 per group), probably due to the high temperature during the test; these high frequencies of crashed piglets would explain the failure to detect significant differences between groups in terms of general mortality (both global and at maternity).

The quantity of supplemented colostrum could be insufficient for creating a significant difference between groups. Plasma IgG concentrations reached a plateau when colostrum intake increased beyond 200 g to 250 g in piglets [1]. This plateau probably reflects the cessation of intact immunoglobulin absorption since colostrum itself will play an important role in the induction of intestinal closure (cessation of intestinal absorption of large molecules) [19]. Furthermore, pre-weaning survival was influenced by numerous factors not accounted for in this study (birth weight, litter size, length of farrowing and dystocia, birth order, room temperature, nutritional status, gender, and maternal and piglet behaviour) [44]. For the smallest piglets, the small sample size could be the cause of the failure to detect significant differences between groups.

## 5. Conclusions

Supplementation with colostrum from multiparous sows led to increased homogeneity in weight and ADG, reducing the variability of final weight and daily growth. In addition, supplemented piglets showed a significant decrease in the use of antibiotics, especially during the nursery phase. Although colostrum supplementation did not reduce the mortality rate significantly, the number of deaths from diarrhoea (the main cause of illness) significantly decreased. The supplemented piglets showed a better immune response to PRRS and influenza (day 60) and *M. hyopneumoniae* (day 21). In the smallest piglets, colostrum supplementation seemed to have a more important effect on mean weight on the first day of life and ADG until day 21. These smallest piglets showed reduced antibiotic treatment use when supplemented. At least in the smallest piglets, colostrum supplementation could improve piglets´ performance and health, although it requires increased time and labour in maternity.

## Figures and Tables

**Table 1:** Reproductive parameters of the gilts from both groups (raw data). Data are means/litter ± SD (standard deviation). CON: control group; SUP: colostrum supplemented group.

Parameter	Group	
	CON	SUP
n (Litters)	18	16
Total piglets	13.5 ± 3.57	14.2 ± 1.98
Born alive	12.4 ± 3.81	13.3 ± 2.12
Stillbirth	0.8 ± 0.81	0.7 ± 0.84
Mummified	0.4 ± 0.85	0.2 ± 0.58
Death at birth	0.7 ± 0.84	0.4 ± 0.51
Total weaned	11.1 ± 0.94	11.2 ± 1.17
Analysed weaned	9.3 ± 2.63	10.6 ± 1.63

**Table 2:** Weights (kg) for survivor piglets for days of age 0–60 from the CON (*n* = 166) and SUP (*n* = 159) groups. SD: Standard deviation. CV: Coefficient of variation. CON: control group; SUP: colostrum supplemented group.

Day	CON		SUP		Variance Comparison (*p*-Value)	ANCOVA Effects (*p*-Values)		
	Mean ± SD	CV	Mean ± SD	CV		Group	Litter	Previous Weight (Covariate)
0	1.3 ± 0.27	0.20	1.3 ± 0.26	0.20	0.761	0.661	<0.001	-----
1	1.4 ± 0.30	0.21	1.4 ± 0.29	0.20	0.696	0.178	<0.001	Day 0: <0.001
10	2.4 ± 0.68	0.28	2.5 ± 0.56	0.22	0.075	0.293	<0.001	Day 0: 0.310
								Day 1: <0.001
21	4.2 ± 1.34	0.31	4.5 ± 1.07	0.24	0.005	0.768	<0.001	Day 0: 0.530
								Day 1: 0.446
								Day 10: <0.001
60	14.2 ± 3.38	0.24	15.2 ± 2.68	0.18	0.012	0.407	<0.001	Day 0: 0.047
								Day 1: 0.678
								Day 10: <0.001
								Day 21: <0.001

**Table 3:** Average daily gain (ADG: kgd^−1^) for survivor piglets for several time intervals from the CON (*n =* 166) and SUP (*n =* 159) groups. SD: Standard deviation. CV: Coefficient of variation. CON: control group; SUP: colostrum supplemented group.

Time Interval (Days)	CON		SUP		Variance Comparison (*p*-Value)	ANCOVA Effects (*p*-Values)		
	Mean ± SD	CV	Mean ± SD	CV		Group	Litter	Previous Weight
0–1	0.07 ± 0.097	1.31	0.11 ± 0.116	1.11	0.594	0.178	<0.001	Day 0: 0.787
0–10	0.11 ± 0.055	0.52	0.12 ± 0.047	0.39	0.038	0.190	<0.001	Day 0: <0.001
0–21	0.14 ± 0.058	0.42	0.15 ± 0.046	0.30	0.003	0.123	<0.001	Day 0 <0.001
0–60	0.21 ± 0.054	0.25	0.23 ± 0.044	0.19	0.026	0.080	<0.001	Day 0: <0.001
1–10	0.11 ± 0.056	0.52	0.12 ± 0.051	0.42	0.195	0.293	<0.001	Day 1: 0.137
10–21	0.17 ± 0.071	0.42	0.18 ± 0.061	0.33	0.056	0.768	<0.001	Day 10: <0.001
21–60	0.26 ± 0.065	0.25	0.27 ± 0.056	0.20	0.148	0.407	<0.001	Day 21: 0.976

**Table 4:** Weights (kg) for survivor piglets under Q1 birth weight (1.100 kg) for days of age 0–60 from CON (*n =* 30) and SUP (*n =* 35) groups. SD: Standard deviation. CV: Coefficient of variation. CON: control group; SUP: colostrum supplemented group.

Day	CON		SUP		Variance Comparison (*p*-Value)	ANCOVA Effects (*p*-Values)		
	Mean ± SD	CV	Mean ± SD	CV		Group	Litter	Previous Weight (Covariate)
0	0.95 ± 0.138	0.15	0.97 ± 0.114	0.12	0.211	0.240	0.248	-----
1	0.99 ± 0.162	0.16	1.05 ± 0.126	0.12	0.138	0.009	0.118	Day 0: <0.001
10	1.70 ± 0.419	0.25	2.03 ± 0.495	0.20	0.863	0.075	0.074	Day 0: 0.144
								Day 1: 0.006
21	3.03 ± 0.864	0.28	3.81 ±0.870	0.23	0.651	0.586	0.061	Day 0: 0.140
								Day 1: 0.244
								Day 10: <0.001
60	10.66 ± 2.51	0.23	14.17 ± 2.687	0.19	0.877	0.060	0.006	Day 0: 0.334
								Day 1: 0.061
								Day 10: 0.728
								Day 21: 0.009

**Table 5:** Average daily gain (ADG: kgd^−1^) for survivor piglets under Q1 (1.100 kg) birth weight for several time intervals from the CON (*n =* 30) and SUP (*n =* 35) groups. SD: Standard deviation. CV: Coefficient of variation. CON: control group; SUP: colostrum supplemented group.

Time Interval (Days)	CON		SUP		Variance Comparison (*p*-Value)	ANCOVA Effects (*p*-Values)		
	Mean ± SD	CV	Mean ± SD	CV		Group	Litter	Previous Weight (Covariate)
0–1	0.05 ± 0.061	1.35	0.08 ± 0.047	0.59	0.766	0.028	0.187	Day 0:0.910
0–10	0.08 ± 0.035	0.47	0.11 ± 0.037	0.35	0.651	0.003	0.049	Day 0: 0.672
0–21	0.10 ± 0.039	0.39	0.14 ± 0.040	0.30	0.731	0.007	0.121	Day 0: 0.383
0–60	0.16 ± 0.041	0.25	0.22 ± 0.044	0.20	0.973	0.068	0.004	Day 0: 0.017
1–10	0.08 ± 0.037	0.46	0.11 ± 0.039	0.36	0.597	0.122	0.075	Day 1: 0.042
10–21	0.12 ± 0.048	0.40	0.16 ± 0.052	0.32	0.945	0.928	0.289	Day 10: 0.001
21–60	0.20 ± 0.052	0.26	0.27 ± 0.055	0.20	0.971	0.254	<0.001	Day 21: 0.092

**Table 6:** Percentages of injectable antibiotic treatment in the CON and SUP groups. Data are count/*n*; percentages are shown in brackets. CON: control group; SUP: colostrum supplemented group. Comparisons between groups were made by using Pearson’s Chi-squared test.

Variable	Period	CON	SUP	*p*-Value
		Count/*n* (%)	Count/*n* (%)	
Initial piglets		99/200 (49.5%)	67/201 (33.3%)	<0.001
	Lactation	31/99 (31.3%)	28/67 (41.8%)	0.373
	Nursery	28/99 (28.3%)	17/67 (25.4%)	
	Both	40/99 (40.4%)	22/67 (32.8%)	
Survivor piglets		78/159 (49.1%)	57/166 (34.3%)	0.010

**Table 7:** Percentages of injectable antibiotic treatment for piglets under Q1 (1.100 kg) in CON and SUP groups. Data are count/n; percentages are shown in brackets. CON: control group; SUP: colostrum supplemented group. Comparisons between groups were made by using Pearson’s Chi-squared test.

Variable	CON	SUP	*p*-Value
	Count/*n* (%)	Count/*n* (%)	
Initial piglets	35/50 (70.0%)	27/52 (51.9%)	0.030
Survivor piglets	23/30 (76.7%)	21/35 (60.0%)	0.243

**Table 8:** Disease distribution in the CON and SUP groups. Data are count/n; percentages are shown in brackets. ^a,b^: different letters in the same row mean significant differences (*p* < 0.05). CON: control group; SUP: colostrum supplemented group. Comparisons between groups were made by using Pearson’s Chi squared test. General symptoms refer to nonspecific symptoms that do not point to any specific disease. Delayed growth indicates narrow animals or lighter than their peers.

Disease	CON	SUP
	Count/*n* (%)	Count/*n* (%)
Polyarthritis	3/110 (2.7%) ^a^	5/67 (7.46%) ^a^
Diarrhoea	68/110 (61.8%) ^a^	25/67 (37.31%) ^b^
General symptoms-delayed growth	39/110 (35.5) ^a^	37/67 (55.22%) ^b^

**Table 9:** Presence of antibodies against PRRS, influenza, and M. hyopneumoniae in 10 individual blood samples from the CON and SUP groups. CON: control group; SUP: colostrum supplemented group. Comparisons between groups were made by using Fisher´s exact test.

Disease	Time	CON	SUP	*p*-Value
	(Day)	Positive Samples	Positive Samples	
PRRS				
	21	0/10 (0%)	4/10 (40%)	0.087
	60	0/10 (0%)	5/10 (50%)	0.033
Influenza				
	21	1/10 (10%)	2/10 (20%)	1.000
	60	0/10 (0%)	5/10 (50%)	0.033
*M. hyopneumoniae*				
	21	0/10 (0%)	5/10 (50%)	0.033
	60	0	0	-

**Table 10:** General mortality and mortality by pathological cause (global and by age) and mortality by diarrhoea (global) in the CON and SUP groups. CON: control group; SUP: colostrum supplemented group. Comparisons between groups were made by using Pearson’s Chi-squared test. Fisher’s exact test was used for mortality by diarrhoea.

Variable	Period	CON	SUP	*p*-Value
		Count/*n* (%)	Count/*n* (%)	
General mortality				
	Global	41/200 (20.5%)	35/201 (17.4%)	0.508
	Lactation	34/200 (17.0%)	32/201 (15.9%)	0.875
	Nursery	7/166(4.2%)	3/169 (1.8%)	0.216
Mortality by pathological causes				
	Global	19/41 (46.3%)	13/35 (37.1%)	0.564
	Lactation	12/34 (35.3%)	10/32 (31.3%)	0.931
	Nursery	7/7(100%)	3/3 (100%)	----
Mortality by diarrhoea				
	Global	15/41(36.6%)	5/35 (14.3%)	0.037

**Table 11:** General mortality and mortality by pathological cause (global and by age) and mortality by diarrhoea (global) in piglets under Q1 birth weight from the CON (*n =* 50) and SUP (*n =* 52) groups. CON: control group; SUP: colostrum supplemented group. Comparisons between groups were made by using Pearson’s Chi-squared test. Fisher’s exact test was used for mortality by diarrhoea.

Variable	Period	CON	SUP	*p*-Value
		Count/*n* (%)	Count/*n* (%)	
General mortality				
	Global	20/50 (40.0%)	17/52 (32.7%)	0.575
	Maternity	16/50 (32.0%)	15/52 (28.8%)	0.896
	Nursery	4/34 (11.8%)	2/37 (5.4%)	0.592
Mortality by pathological causes				
	Global	11/20 (55.0%)	7/17 (41.2%)	0.611
	Maternity	7/16 (43.8%)	5/15 (33.3%)	0.821
	Nursery	4/4(100%)	2/2 (100%)	
Mortality by diarrhoea				
	Global	7/20 (35.0%)	3/17 (17.6%)	0.288

## Data Availability

Data supporting reported results can be sent to anyone interested by contacting the corresponding author.
